# Prognostic value of heart rate variability for risk of serious adverse events in continuously monitored hospital patients

**DOI:** 10.1007/s10877-024-01193-8

**Published:** 2024-08-20

**Authors:** Nikolaj Aagaard, Markus Harboe Olsen, Oliver Wiik Rasmussen, Katja K. Grønbaek, Jesper Mølgaard, Camilla Haahr-Raunkjaer, Mikkel Elvekjaer, Eske K. Aasvang, Christian S. Meyhoff

**Affiliations:** 1 Department of Anaesthesia and Intensive Care, Copenhagen University Hospital – Bispebjerg and Frederiksberg, Copenhagen, Denmark; 2 Department of Neuroanaesthesiology, The Neuroscience Centre, Copenhagen University Hospital - Rigshospitalet, Copenhagen, Denmark; 3 Biomedical Engineering, Department of Health Technology, Technical University of Denmark, Lyngby, Denmark; 4 Department of Anaesthesia, CKO, Copenhagen University Hospital – Rigshospitalet, Copenhagen, Denmark; 5 Department of Clinical Medicine, University of Copenhagen, Copenhagen, Denmark

**Keywords:** Heart rate variability, Serious adverse events, Acute medical disease, Major surgery, Continuous vital sign monitoring

## Abstract

Technological advances allow continuous vital sign monitoring at the general ward, but traditional vital signs alone may not predict serious adverse events (SAE). This study investigated continuous heart rate variability (HRV) monitoring’s predictive value for SAEs in acute medical and major surgical patients. Data was collected from four prospective observational studies and two randomized controlled trials using a single-lead ECG. The primary outcome was any SAE, secondary outcomes included all-cause mortality and specific non-fatal SAE groups, all within 30 days. Subgroup analyses of medical and surgical patients were performed. The primary analysis compared the last 24 h preceding an SAE with the last 24 h of measurements in patients without an SAE. The area under a receiver operating characteristics curve (AUROC) quantified predictive performance, interpretated as low prognostic ability (0.5–0.7), moderate prognostic ability (0.7–0.9), or high prognostic ability (> 0.9). Of 1402 assessed patients, 923 were analysed, with 297 (32%) experiencing at least one SAE. The best performing threshold had an AUROC of 0.67 (95% confidence interval (CI) 0.63–0.71) for predicting cardiovascular SAEs. In the surgical subgroup, the best performing threshold had an AUROC of 0.70 (95% CI 0.60–0.81) for neurologic SAE prediction. In the medical subgroup, thresholds for all-cause mortality, cardiovascular, infectious, and neurologic SAEs had moderate prognostic ability, and the best performing threshold had an AUROC of 0.85 (95% CI 0.76–0.95) for predicting neurologic SAEs. Predicting SAEs based on the accumulated time below thresholds for individual continuously measured HRV parameters demonstrated overall low prognostic ability in high-risk hospitalized patients. Certain HRV thresholds had moderate prognostic ability for prediction of specific SAEs in the medical subgroup.

**Supplementary Information:**

The online version contains supplementary material available at 10.1007/s10877-024-01193-8.

## Introduction

Late recognition of deteriorating patients is a major problem in hospital wards, contributing to in-hospital mortality and intensive care unit admissions [1, 2]. Several countries have implemented routine manual monitoring of vital signs at fixed intervals such as the National Early Warning Score (NEWS) [3, 4]. However, clinical deterioration also occurs between observations, which might explain the lack of documented effect of NEWS on morbidity and mortality [5–7]. Thus, early recognition is crucial to allow for timely treatment and potentially prevent further deterioration. Recent technological advances allow continuous vital sign monitoring at the general ward that may detect early clinical deterioration and thereby decrease the risk of serious adverse events (SAE) [8], but traditional vital signs may not consistently predict imminent SAEs [9, 10]. This technology also enables continuous evaluation of heart rate variability (HRV), which is determined from variations in electrocardiographic (ECG) R-R intervals and considered an important indicator of autonomic nervous system (ANS) activity [11]. HRV can be affected by physical, mental, and surgical stress in a clinical setting [12, 13]. HRV is often altered in elderly patients and patients with chronic or acute diseases [14]. A reduction in autonomic regulation of heart rate, resulting in lower 24-h measurements of the standard deviation of normal-to-normal R-R intervals (SDNN) and root mean square differences of successive R-R intervals (RMSSD), is associated with adverse cardiac events and mortality [15–18]. Consequently, the utilization of HRV alerts, which offer a more refined assessment of autonomic functions, potentially holds substantial predictive value [14]. As an example, HRV depression, defined as a decrease in R-R interval variation and thus lower RMSSD, is a marker of increased sympathetic tone and stress, associated with poor outcomes in patients with sepsis [19–22]. As complications can arise without clinical recognition in the general ward [23, 24], deviations in HRV monitored with continuous wireless monitoring systems may serve as a prognostic marker of upcoming critical complications.

Previous studies have generally investigated long term outcomes in specific medical populations and focused on predefined thresholds for a limited number of HRV parameters, without systematically evaluating multiple thresholds across various parameters [14, 18, 25–27]. This study aimed at exploring the prognostic ability of HRV-derived measures for subsequent SAEs when measured continuously with a wearable single-lead ECG device in patients hospitalized for acute medical disease or after major surgery. We investigated the predictive ability of several HRV parameters for SAEs by calculating the area under the curve (AUC) based on the last 24 h of continuous monitoring before the occurrence of SAEs.

## Methods

### Included studies

Data were collected from four prospective observational studies (NCT03660501, NCT03491137, NCT04473001, and NCT04628858) and two randomized controlled trials (NCT04661748 and NCT04640415). All studies were approved by the Danish Data Protection Agency (P-2019-690) and regional ethics committee (H-20033246, H-20034555, H-18026653, H-17033535, H-20002220, and H-19086583). One trial was ongoing at the time of data extraction (NCT04661748), and only patients with registered outcomes were included. All studies’ inclusion and exclusion criteria are listed in Supplemental Table 1.

### Data collection

Data were collected using the clinically validated CE- and FDA-approved Isansys Lifetouch (Isansys Lifecare, Oxfordshire, United Kingdom) [28, 29]. Isansys Lifetouch is an ECG patch attached to the patient’s chests with two electrodes placed 15 cm apart over the anterior left aspect of the patient’s thorax at an angle of 45° with its base in the fourth intercostal space approximately 2 cm lateral to the sternum. The device recorded a single lead ECG and registered heart and respiratory rates. ECG segments of approximately 10 s each minute were transferred and saved directly via Bluetooth to the Isansys gateway. When patients were out of range for Bluetooth connection, data were stored automatically on the device and transferred when the connection was re-established. Patient demographic data were collected including height, weight, age, smoking status, and pre-existing medical conditions through the Charlson Comorbidity Index [30]. Informed consent was obtained from all patients included in the study.

### Exposures

Exposure variables were accumulated time in minutes below specific thresholds for each of the different HRV-derived measures (*see HRV-derived measures)* in the last 24 h of measuring before the first SAE. If no SAE occurred, the last 24 h of HRV measurements in each patient were used for the primary analysis. Up to 1000 thresholds for each measure were investigated for the prognostic ability (*see* *Statistical analysis*). Only patients with at least 18 h of ECG monitoring in the 24 h of interest were included in the analysis. Patients were excluded from the primary analysis if an SAE occurred within 18 h after initiating monitoring to give patients with and without SAE similar exposure time.

The secondary analysis included only patients with an SAE after at least 48 h of measurements. We aimed to compare the last 24 h of measurements before an SAE to the period measured 24 to 48 h prior to the SAE. We conducted this analysis to determine if HRV changes during monitoring could predict development of SAEs. Thus, the primary analysis assessed whether HRV differed from a control group without SAEs, while the secondary analysis investigated if HRV changed in the period leading up to an SAE compared to earlier monitoring.

A tertiary analysis compared the first 24 h to the last 24 h of HRV measurements in patients without an SAE, to investigate differences between the start and end of the monitoring period. This analysis is relevant as a wide range of factors can alter HRV [14]. Given that all study participants were admitted for major surgery or acute medical conditions, a degree of HRV alterations are expected [14]. This analysis aimed to evaluate changes in HRV during the monitoring period in patients who did not develop an SAE.

The same requirements for missing data were applied to all the analyses and only HRV measurements preceding the first SAE were included to avoid bias of vital signs and ECG deviations resulting from the first SAE and medical interventions to correct it.

#### HRV-derived measures

HRV was evaluated using time-domain and frequency-domain measures [14]. Time-domain measures were used to quantify variability in the time between successive heartbeats, and included SDNN, RMSSD, the standard deviation of successive R-R interval differences (SDSD), the percentage of adjacent R-R intervals that differ from each other by more than 50 ms (pNN50), and the mean of R-R intervals (RRMean), with higher values reflecting greater variation in R-R intervals [14].

Frequency-domain measures can be calculated as absolute power or normalized power for different frequency bands including very-low-frequency (vLF, ≤ 0.04 Hz); low-frequency (LF, 0.04–0.15 Hz); and high-frequency (HF, 0.15–0.4 Hz) [14]. Power is defined as signal energy within a frequency band with the absolute power of a frequency band calculated as milliseconds squared divided by cycles per second [14]. Normalized power was reported in normalized units (n.u.), calculated as the absolute power of the specific frequency band divided by the summed absolute power of the vLF, LF, and HF bands. The normalized R-R interval power of the vLF, LF, and HF bands was included for statistical analysis [31].

24-h measurements of SDNN are considered the gold standard for medical stratification of cardiac risk and prediction of both morbidity and mortality, hence SDNN was the primary exposure variable [11, 14]. Time-domain and frequency-domain measures were calculated for one-minute intervals and are defined in Supplemental Table 2 [14].

### Outcomes

The primary outcome was any SAE within 30 days. SAEs were defined as any medical life-threatening complication, resulting in death, hospitalization, prolongation of existing hospitalization, or significant disability according to the International Conference on Harmonisation—Good Clinical Practice guideline [32]. The secondary outcomes were all-cause mortality and non-fatal SAEs within the following classifications: cardiovascular, respiratory, infectious, neurologic, and other SAEs. All outcomes were based upon a standardized outcome manual including international defined criteria, such as acute renal failure, myocardial infarction, and sepsis [33–35].

### Statistical analysis

Descriptive statistics were used to analyse baseline characteristics and frequency of patients with and without SAEs. Categorical variables are presented as numbers with percentages and continuous variables as medians with interquartile ranges.

For each individual HRV parameter, we calculated the accumulated time below the threshold, for up to 1000 different thresholds. As all patients had an accumulated time below the various thresholds this was used to evaluate the prognostic ability of each individual HRV-parameter. We calculated the area under a receiver operating characteristics curve (AUROC) and the corresponding 95% confidence interval (CI), using stratified bootstrapping, with the pROC-package [36]. The binary response variable was the occurrence of SAEs, and the continuous predictor variable was the accumulated time below each specific threshold. These calculations were repeated across all thresholds for each individual HRV parameter. AUROC quantified the prognostic ability, and was interpreted as representing ‘no better than chance’ (~ 0.5), low prognostic ability (0.5–0.7), moderate prognostic ability (0.7–0.9), and high prognostic ability (> 0.9) [37].

Additional calculations of the optimal cut-off and corresponding sensitivity and specificity were performed for thresholds with a lower 95% CI of moderate prognostic ability or above (AUROC > 0.7); and if none had a lower 95% CI above moderate prognostic ability, the calculations were performed for the threshold of each HRV parameter that achieved the highest AUROC. This was performed with both the primary outcome and each specific group of secondary outcomes as the binary response variable. The optimal cut-offs were defined as the values of the predictor variable, that maximize Youden’s Index, i.e. the maximum sum of specificity and sensitivity [38].

For the primary outcome of any SAE, the threshold with the highest AUROC for each HRV parameter, along with the specific HRV parameter that had the highest AUROC for each specific secondary outcome are presented in tables with the AUC, the corresponding 95% CI, the threshold, the optimal cut-off, and the corresponding sensitivity and specificity.

Subgroup analyses were performed for medical patients and surgical patients. All statistical analyses were performed using the statistical software R version 4.2.1 (R Core Team, Vienna, Austria).

## Results

**Fig. 1:**
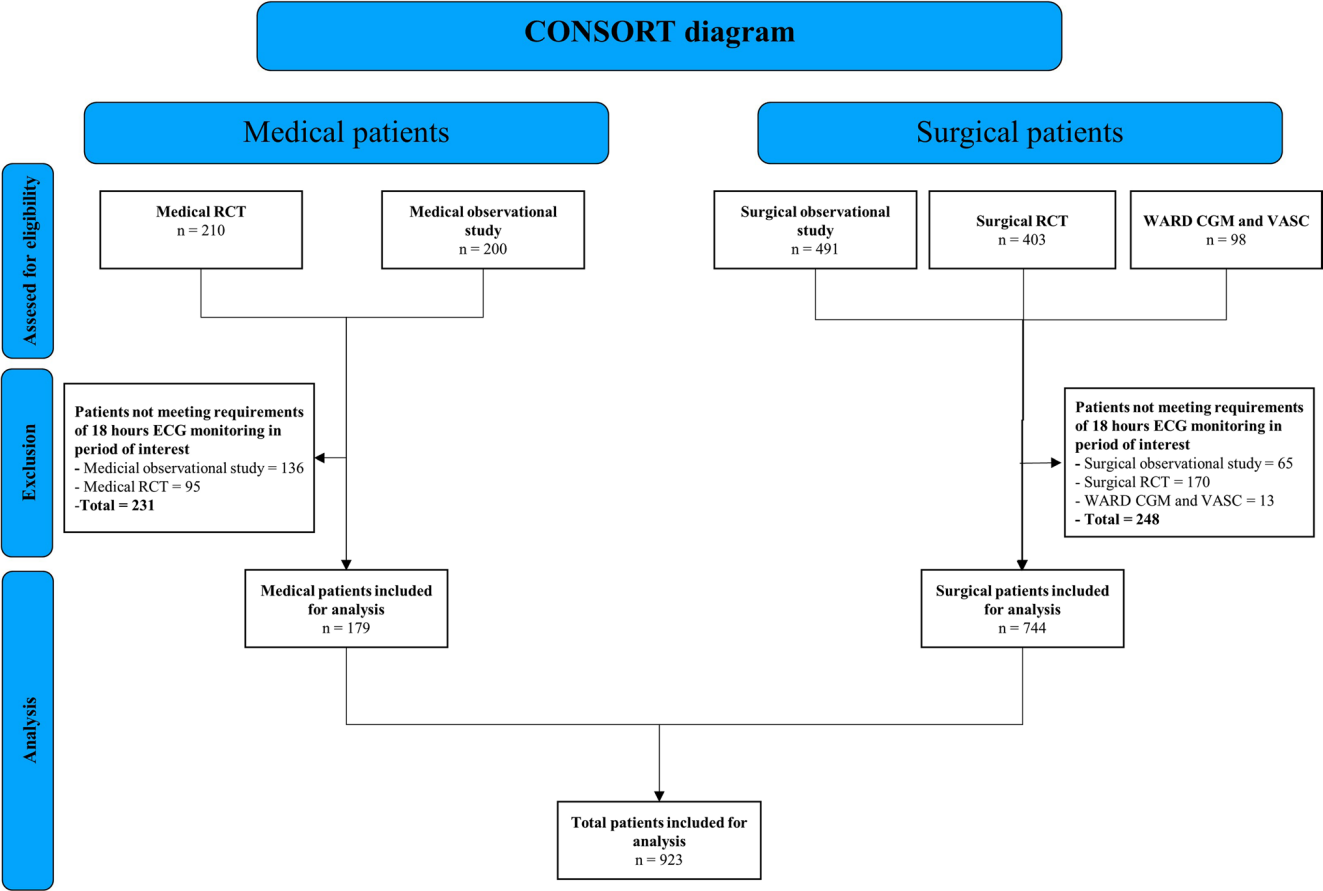
CONSORT diagram of patient inclusion and data analysis. *RCT* randomized controlled trial, *WARD* Wireless Assessment of Respiratory and Circulatory Distress, *CGM* continuous glucose monitoring, *VASC* vascular surgery

A total of 1402 patients from six studies and trials were assessed for eligibility. A total of 923 patients were included for analysis, while 479 were excluded as the requirement for duration of recording was not met (Fig. 1), and 297 included patients (32%) had one or more SAEs. There were 27 instances of all-cause mortality, 30 cardiovascular SAEs, 45 respiratory SAEs, 72 infectious SAEs, 9 neurologic SAEs, and 114 other SAEs. The baseline characteristics of the patients are presented in Table 1.

**Table 1:** Baseline characteristics

Characteristic	No SAE within 30 days N = 626	SAE within 30 days N = 297
Sex		
Female, no. (%)	259 (41.8)	100 (34)
Age, yes median [IQR]	70 [64 - 76]	71 [65 - 75]
Surgical admission, no. (%)	477 (76.3)	258 (86.9)
Medical admission, no. (%)	143 (22.7)	36 (12.1)
Smoking status, no. (%)		
Never	163 (26.5)	78 (26.5)
Former	336 (54.4)	171 (58.2)
Current	118 (19.2)	45 (15.3)
ASA score, no. (%)		
I	23 (4.8)	6 (2.3)
II	255 (53.5)	104 (40.3)
III	197 (41.3)	145 (56.2)
IV	2 (0.4)	3 (1.2)
Body mass index, kg/m^2^ median [IQR]	25 [23 - 29]	25 [23 - 29]
Charlson comorbidity index score, no. (%)		
0	24 (3.9)	1 (0.3)
1–2	45 (7.3)	17 (5.8)
3–4	213 (34.7)	90 (30.7)
5–6	236 (38.5)	124 (42.3)
7–8	57 (9.3)	44 (15)
9 +	39 (6.2)	17 (5.8)
Comorbidities, no. (%)		
Diabetes mellitus	111 (17.9)	74 (25.3)
Chronic pulmonary disease	161 (26.0)	69 (23.5)
Myocardial infarction	21 (3.4)	12 (4.1)
Cerebrovascular disease	51 (8.2)	20 (6.8)
Cancer	403 (65.4)	208 (70.7)
Baseline vital signs, median [IQR]		
Systolic blood pressure, mmHg	137 [124 - 152]	134 [123 - 150]
Diastolic blood pressure, mmHg	76 [67 - 85]	77 [68 - 83]
Peripheral oxygen saturation, %	97 [96 - 99]	98 [96 - 99]
Type of surgery, no. (%)		
Pancreatic resection	92 (19)	85 (32.8)
Esophagus resection	58 (12)	41 (15.8)
Ventricle resection	23 (4.8)	8 (3.1)
Small intestine or colon resection	130 (26.9)	28 (10.8)
Rectum resection	32 (6.6)	21 (8.1)
Other major procedures	141 (29.2)	73 (28.2)
Acute medical admission, no. (%)		
AECOPD	46 (32.2)	18 (50)
Sepsis	4 (2.8)	0
Dyspnoea	59 (41.3)	9 (25.0)
Pneumonia	33 (23.1)	8 (22.2)
Heart failure	1 (0.7)	1 (2.8)

*SAE* serious adverse events,* N*: numbers, *IQR* interquartile range, *ASA* American Society of Anesthesiologists, *AECOPD* acute exacerbation of chronic pulmonary disease

### Primary analysis

**Fig. 2:**
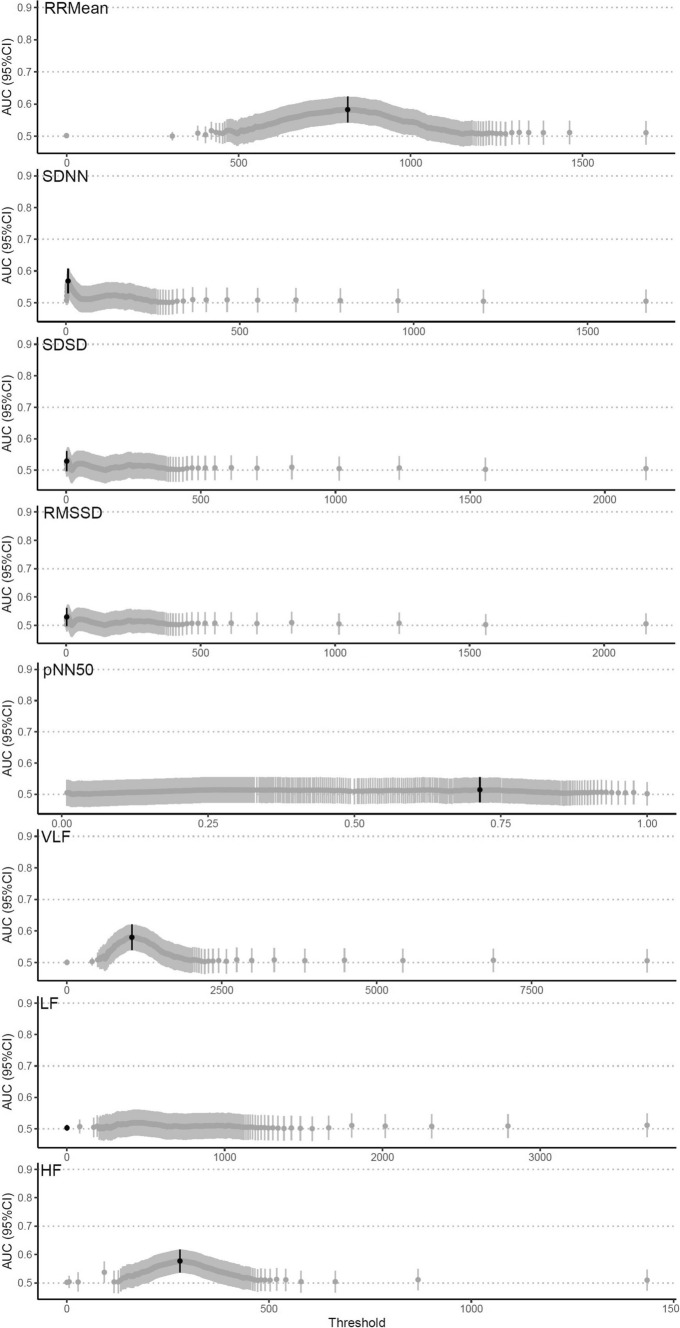
Best performing thresholds in the primary analysis. *AUC* area under the curve, *95% CI* 95% confidence interval, *SDNN* standard deviation of R-R intervals, *RMSSD* root mean square differences of successive R-R intervals, *RRMean* mean of R-R intervals, *SDSD* standard deviation of successive R-R interval differences, *pNN50* percentage of adjacent R-R intervals that differ from each other by more than 50 ms, *HF* high-frequency; 0.15–0.4 Hz, *LF* low-frequency; 0.04–0.15 Hz, *vLF* very-low-frequency; ≤ 0.04 Hz

When comparing the last 24 h before any SAE with the last 24 h of monitoring in those without SAEs, the optimal threshold for the primary exposure variable SDNN demonstrated an AUC of 0.57 (95% CI 0.53–0.61), sensitivity of 0.47 (95% CI 0.41–0.53), and specificity of 0.65 (95% CI 0.62–0.69). Similarly, the other HRV parameters presented with a low prognostic ability (Fig. 2). For the specific outcomes, RMSSD had the largest point estimates of AUC 0.67 (95% CI 0.63–0.71) for predicting cardiovascular SAEs, with a sensitivity and specificity of 0.03 (95% CI 0–0.17) and 0.63 (95% CI 0.59–0.67), respectively (Table 2).

**Table 2:** Best performing thresholds for the primary analysis and secondary analysis

Type of SAE	HRV parameter	Number (control/SAE)	Threshold	AUC (95% CI)	Cut-off	Sensitivity (95% CI)	Specificity (95% CI)
Primary analysis							
Any SAE	LF	923 (626/297)	0.42	0.50 (0.49–0.51)	5.5	0.01 (0–0.03)	1 (0.99–1)
Any SAE	pNN50	923 (626/297)	0.71	0.51 (0.47–0.55)	1291.5	0.69 (0.64–0.75)	0.25 (0.22–0.29)
Any SAE	RMSSD	923 (626/297)	2.35	0.53 (0.50–0.56)	11.5	0.15 (0.11–0.20)	0.93 (0.91–0.95)
Any SAE	SDSD	923 (626/297)	2.34	0.53 (0.50–0.56)	11.5	0.15 (0.11–0.19)	0.93 (0.91–0.95)
Any SAE	SDNN	923 (626/297)	6.24	0.57 (0.53–0.61)	10.5	0.47 (0.41–0.53)	0.65 (0.62–0.69)
Any SAE	HF	923 (626/297)	279.69	0.58 (0.54–0.62)	1000.5	0.54 (0.48–0.60)	0.61 (0.57–0.65)
Any SAE	RRMean	923 (626/297)	816.77	0.58 (0.54–0.62)	1265.5	0.47 (0.41–0.53)	0.69 (0.65–0.72)
Any SAE	VLF	923 (626/297)	1049.16	0.58 (0.54–0.62)	816.5	0.65 (0.60–0.71)	0.48 (0.44–0.52)
All-cause mortality	RRMean	653 (626/27)	896.61	0.65 (0.55–0.76)	1439.5	0.48 (0.29–0.68)	0.80 (0.77–0.83)
Cardiovascular SAE	RMSSD	656 (626/30)	2.80	0.67 (0.63–0.71)	0.5	0.03 (0–0.17)	0.63 (0.59–0.67)
Infectious SAE	HF	698 (626/72)	306.22	0.62 (0.55–0.69)	1342.5	0.50 (0.38–0.62)	0.70 (0.67–0.74)
Infectious SAE	RRMean	698 (626/72)	849.23	0.62 (0.55–0.69)	1288.0	0.61 (0.49–0.72)	0.59 (0.55–0.63)
Infectious SAE	VLF	698 (626/72)	1013.67	0.62 (0.55–0.69)	801.5	0.67 (0.55–0.77)	0.53 (0.49–0.57)
Neurologic SAE	RRMean	635 (626/9)	1041.81	0.64 (0.55–0.73)	1428.5	0 (0–0.34)	0.48 (0.44–0.52)
Respiratory SAE	RRMean	671 (626/45)	841.53	0.59 (0.51–0.67)	805.5	0.89 (0.76–0.96)	0.33 (0.30–0.37)
Other SAE	VLF	740 (626/114)	1265.47	0.59 (0.53–0.65)	1381.5	0.53 (0.43–0.62)	0.64 (0.60–0.68)
Secondary analysis							
Any SAE	pNN50	189 (189/189)	1.00	0.67 (0.61–0.73)	1439.5	0.64 (0.57–0.71)	0.88 (0.83–0.93)
Any SAE	LF	189 (189/189)	3677.32	0.68 (0.63–0.74)	1439.5	0.67 (0.59–0.73)	0.88 (0.83–0.93)
Any SAE	HF	189 (189/189)	1434.58	0.69 (0.63–0.75)	1439.5	0.68 (0.61–0.74)	0.88 (0.83–0.93)
Any SAE	SDNN	189 (189/189)	1668.10	0.69 (0.63–0.75)	1439.5	0.68 (0.61–0.74)	0.88 (0.82–0.92)
Any SAE	VLF	189 (189/189)	9360.91	0.69 (0.64–0.75)	1439.5	0.68 (0.61–0.75)	0.88 (0.82–0.92)
Any SAE	RMSSD	189 (189/189)	2155.71	0.70 (0.64–0.75)	1439.5	0.68 (0.61–0.75)	0.88 (0.82–0.92)
Any SAE	SDSD	189 (189/189)	2152.69	0.70 (0.64–0.75)	1439.5	0.68 (0.61–0.75)	0.88 (0.82–0.92)
Any SAE	RRMean	189 (189/189)	1684.53	0.70 (0.64–0.76)	1439.5	0.70 (0.63–0.76)	0.88 (0.82–0.92)
All-cause mortality	LF	13 (13/13)	1531.80	0.80 (0.60–0.99)	1439.5	0.85 (0.55–0.98)	0.85 (0.55–0.98)
All-cause mortality	RMSSD	13 (13/13)	453.33	0.80 (0.60–0.99)	1439.5	0.85 (0.55–0.98)	0.85 (0.55–0.98)
All-cause mortality	SDSD	13 (13/13)	453.19	0.80 (0.60–0.99)	1439.5	0.85 (0.55–0.98)	0.85 (0.55–0.98)
All-cause_mortality	VLF	13 (13/13)	2247.84	0.80 (0.60–0.99)	1439.5	0.85 (0.55–0.98)	0.85 (0.55–0.98)
Cardiovascular SAE	VLF	15 (15/15)	582.69	0.70 (0.57–0.83)	0.5	0.40 (0.16–0.68)	1 (0.78–1)
Cardiovascular SAE	SDNN	15 (15/15)	14.93	0.70 (0.51–0.90)	142.5	0.13 (0.02–0.40)	0.47 (0.21–0.73)
Cardiovascular SAE	pNN50	15 (15/15)	0.01	0.70 (0.50–0.89)	579.5	0.13 (0.02–0.40)	0.47 (0.21–0.73)
Infectious SAE	pNN50	50 (50/50)	0.99	0.70 (0.59–0.81)	1439.5	0.64 (0.49–0.77)	0.92 (0.81–0.98)
Infectious SAE	RRMean	50 (50/50)	1591.38	0.70 (0.59–0.81)	1439.5	0.66 (0.51–0.79)	0.92 (0.81–0.98)
Neurologic SAE	RRMean	5 (5/5)	392.56	0.07 (0.46–0.94)	2.0	0.40 (0.05–0.85)	1 (0.48–1)
Respiratory SAE	VLF	28 (28/28)	8076.49	0.76 (0.61–0.90)	1439.5	0.71 (0.51–0.87)	0.96 (0.82–1)
Other SAE	RRMean	78 (78/78)	1604.48	0.69 (0.60–0.78)	1439.5	0.71 (0.59–0.80)	0.85 (0.75–0.92)

*SAE* serious adverse events, *HRV* heart rate variability, *AU* area under the curve, *95% CI* 95% confidence interval, *SDNN* standard deviation of R-R intervals, *RMSSD* root mean square differences of successive R-R intervals, *RRMean* mean of R-R intervals, *SDSD* standard deviation of successive R-R interval differences, *pNN50* percentage of adjacent R-R intervals that differ from each other by more than 50 ms, *HF* high-frequency; 0.15–0.4 Hz, *LF* low-frequency; 0.04–0.15 Hz, *vLF* very-low-frequency; ≤ 0.04 Hz

In the surgical subgroup, the best performing thresholds for any SAEs and the specific SAEs had a low prognostic ability. In the medical subgroup, the optimal thresholds for predicting any SAE demonstrated a low prognostic ability. When investigating specific SAEs, multiple thresholds presented with moderate prognostic ability for all-cause mortality, cardiovascular, infectious, and neurologic SAEs. HF demonstrated the largest point estimate of AUC with statistical significance for predicting neurologic SAEs (AUC: 0.85; 95%CI (0.76–0.95). RMSSD and SDSD had the largest point estimates of AUC with statistical significance for predicting cardiovascular SAE (AUC: 0.84; 95%CI 0.73–0.95) (Table 3).

**Table 3:** Best performing thresholds for the primary analysis in the medical and surgical subgroups

Type of SAE	HRV parameter	Number (control/sae)	Threshold	AUC (95% CI)	Cut-off	Sensitivity (95% CI)	Specificity (95% CI)
Medical subgroup							
Any SAE	VLF	179 (143/36)	455.35	0.52 (0.50–0.53)	0.5	0 (0–0.10)	0.97 (0.92–0.99)
Any SAE	RRMean	179 (143/36)	1898.38	0.57 (0.47–0.67)	1417.0	0.69 (0.52–0.84)	0.47 (0.38–0.55)
Any SAE	pNN50	179 (143/36)	0.11	0.58 (0.47–0.69)	405.5	0.44 (0.28–0.62)	0.31 (0.23–0.39)
Any SAE	LF	179 (143/36)	2654.85	0.58 (0.48–0.68)	1414.0	0.69 (0.52–0.84)	0.47 (0.38–0.55)
Any SAE	RMSSD	179 (143/36)	843.76	0.58 (0.48–0.68)	1416.0	0.67 (0.49–0.81)	0.49 (0.41–0.57)
Any SAE	SDSD	179 (143/36)	843.32	0.58 (0.48–0.68)	1416.0	0.67 (0.49–0.81)	0.49 (0.41–0.57)
Any SAE	SDNN	179 (143/36)	472.12	0.58 (0.48–0.69)	1410.0	0.67 (0.49–0.81)	0.48 (0.39–0.56)
Any SAE	HF	179 (143/36)	190.26	0.60 (0.50–0.70)	84.5	0.56 (0.38–0.72)	0.67 (0.59–0.75)
All-cause mortality	RMSSD	148 (143/5)	17.41	0.77 (0.63–0.90)	25.5	0.2 (0.01–0.72)	0.22 (0.16–0.30)
All-cause mortality	SDSD	148 (143/5)	17.40	0.77 (0.63–0.90)	25.5	0.2 (0.01–0.72)	0.22 (0.16–0.30)
Cardiovascular SAE	RMSSD	148 (143/5)	15.93	0.84 (0.73–0.95)	73.0	0 (0–0.52)	0.34 (0.26–0.42)
Cardiovascular SAE	SDSD	148 (143/5)	16.00	0.84 (0.73–0.95)	75.0	0 (0–0.52)	0.34 (0.26–0.42)
Cardiovascular SAE	pNN50	148 (143/5)	0.03	0.83 (0.73–0.93)	116.0	0 (0–0.52)	0.30 (0.23–0.38)
Cardiovascular SAE	LF	148 (143/5)	219.86	0.78 (0.74–0.82)	0.5	0 (0–0.52)	0.45 (0.36–0.53)
Infectious SAE	RMSSD	146 (143/3)	3.23	0.78 (0.56–1)	0.5	1 (0.29–1)	0.57 (0.49–0.66)
Infectious SAE	SDSD	146 (143/3)	3.23	0.78 (0.56–1)	0.5	1 (0.29–1)	0.58 (0.50–0.66)
Neurologic SAE	HF	146 (143/3)	174.07	0.85 (0.76–0.95)	56.0	1 (0.29–1)	0.78 (0.71–0.85)
Neurologic SAE	RMSSD	146 (143/3)	3.94	0.77 (0.73–0.81)	0.5	0 (0–0.71)	0.46 (0.38–0.55)
Neurologic SAE	SDSD	146 (143/3)	3.94	0.77 (0.73–0.81)	0.5	0 (0–0.71)	0.46 (0.38–0.55)
Respiratory SAE	pNN50	156 (143/13)	0.80	0.62 (0.48–0.76)	1417.5	0.62 (0.32–0.86)	0.64 (0.55–0.72)
Respiratory SAE	RRMean	156 (143/13)	780.01	0.62 (0.46–0.78)	949.5	0.85 (0.55–0.98)	0.43 (0.34–0.51)
Respiratory SAE	HF	156 (143/13)	251.24	0.62 (0.44–0.80)	483.5	0.92 (0.64–1)	0.35 (0.27–0.43)
Other SAE	HF	150 (143/7)	186.14	0.64 (0.44–0.85)	93.5	0.57 (0.18–0.90)	0.73 (0.65–0.80)
Surgical subgroup							
Any SAE	pNN50	744 (483/261)	0.71	0.53 (0.49–0.58)	1367.5	0.61 (0.55–0.67)	0.30 (0.26–0.34)
Any SAE	SDSD	744 (483/261)	2.35	0.53 (0.50–0.57)	11.5	0.16 (0.12–0.21)	0.92 (0.89–0.94)
Any SAE	LF	744 (483/261)	423.67	0.54 (0.49–0.58)	661.5	0.40 (0.34–0.46)	0.69 (0.65–0.73)
Any SAE	RMSSD	744 (483/261)	2.36	0.54 (0.50–0.57)	11.5	0.16 (0.12–0.22)	0.92 (0.90–0.95)
Any SAE	SDNN	744 (483/261)	6.20	0.58 (0.54–0.62)	11.5	0.46 (0.39–0.52)	0.70 (0.66–0.74)
Any SAE	HF	744 (483/261)	279.36	0.60 (0.56–0.64)	981.5	0.54 (0.48–0.61)	0.65 (0.60–0.69)
Any SAE	VLF	744 (483/261)	1003.52	0.60 (0.56–0.64)	671.5	0.64 (0.58–0.70)	0.55 (0.51–0.60)
Any SAE	RRMean	744 (483/261)	820.11	0.61 (0.56–0.65)	829.5	0.71 (0.65–0.77)	0.46 (0.42–0.51)
All-cause mortality	RRMean	505 (483/22)	817.22	0.69 (0.57–0.8)	1279.5	0.64 (0.41–0.83)	0.72 (0.68–0.76)
Cardiovascular SAE	SDSD	508 (483/25)	2.92	0.68 (0.63–0.73)	0.5	0.08 (0.01–0.26)	0.58 (0.53–0.62)
Infectious SAE	HF	552 (483/69)	281.02	0.64 (0.57–0.71)	939.5	0.64 (0.51–0.75)	0.6 (0.55–0.64)
Infectious SAE	RRMean	552 (483/69)	831.18	0.64 (0.57–0.71)	873.5	0.78 (0.67–0.87)	0.46 (0.41–0.5)
Neurologic SAE	RRMean	489 (483/6)	1041.50	0.70 (0.60–0.81)	1427.5	0 (0–0.46)	0.45 (0.4–0.49)
Respiratory SAE	RRMean	515 (483/32)	895.78	0.60 (0.51–0.69)	1100.0	0.91 (0.75–0.98)	0.39 (0.34–0.43)
Other SAE	HF	590 (483/107)	258.92	0.60 (0.54–0.66)	544.0	0.62 (0.52–0.71)	0.59 (0.55–0.64)
Other SAE	RRMean	590 (483/107)	710.19	0.60 (0.54–0.66)	675.0	0.43 (0.33–0.53)	0.76 (0.72–0.8)
Other SAE	VLF	590 (483/107)	1110.25	0.60 (0.54–0.66)	1239.0	0.54 (0.44–0.64)	0.65 (0.61–0.7)

*SAE* serious adverse events, *HRV* heart rate variability, *AUC* area under the curve, *95% CI* 95% confidence interval, *SDNN* standard deviation of R-R intervals, *RMSSD* root mean square differences of successive R-R intervals, *RRMean* mean of R-R intervals, *SDSD* standard deviation of successive R-R interval differences, *pNN50* percentage of adjacent R-R intervals that differ from each other by more than 50 ms, *HF* high-frequency; 0.15–0.4 Hz, *LF* low-frequency; 0.04–0.15 Hz, *vLF* very-low-frequency; ≤ 0.04 Hz

### Secondary analysis

**Fig. 3:**
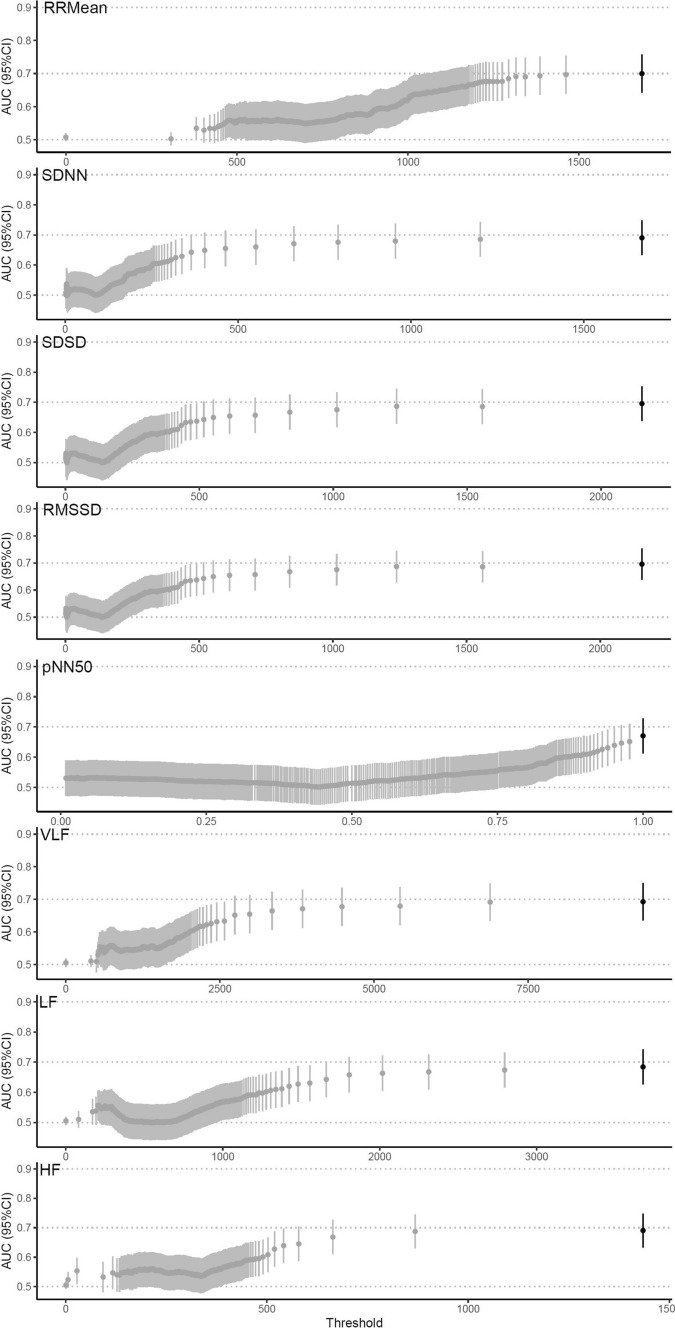
Best performing thresholds in the secondary analysis. *AUC* area under the curve, *95% CI* 95% confidence interval, *SDNN* standard deviation of R-R intervals, *RMSSD* root mean square differences of successive R-R intervals, *RRMean* mean of R-R intervals, *SDSD* standard deviation of successive R-R interval differences, *pNN50* percentage of adjacent R-R intervals that differ from each other by more than 50 ms, *HF* high-frequency; 0.15–0.4 Hz, *LF* low-frequency; 0.04–0.15 Hz, *vLF* very-low-frequency; ≤ 0.04 Hz

When comparing the last 24 h with the 24 to 48 h of measurements before an SAE, the best performing threshold for RRMean, demonstrated the largest point estimate of AUC 0.70 (95% CI 0.64–0.76), with sensitivity and specificity of 0.70 (95% CI 0.63–0.76) and 0.88 (95% CI 0.82–0.92), respectively (Fig. 3). RMSSD and SDSD also had moderate prognostic ability with AUC 0.70 (95% CI 0.64–0.75). The remaining HRV parameters for any SAEs presented with a low prognostic ability. The best performing thresholds for specific SAEs all presented with point estimates of moderate prognostic ability, except for other SAEs. LF, RMSSD, SDSD, and VLF presented with the largest point estimates of AUC for predicting all-cause mortality (AUC:0.8; 95%CI 0.60–0.99) (Table 2).

As most patients included for the secondary analysis were surgical patients, the results in this subgroup were nearly identical with similar HRV parameters demonstrating moderate prognostic ability for any and specific SAEs. For any SAEs in the medical subgroup pNN50 demonstrated the largest point estimate of AUC 0.70 (95% CI 0.50–0.90) with sensitivity and specificity of 0.61 (95% CI 0.36–0.83) and 1.0 (95% CI 0.81–1.00), respectively. The remaining HRV parameters demonstrated a low prognostic ability for any SAE prediction. For the specific SAEs, the best performing thresholds demonstrated moderate or high prognostic ability, but none had a lower confidence limit > 0.7. Detailed subgroup results for the secondary analysis are presented in Supplemental Table 3.

### Tertiary analysis

**Fig. 4:**
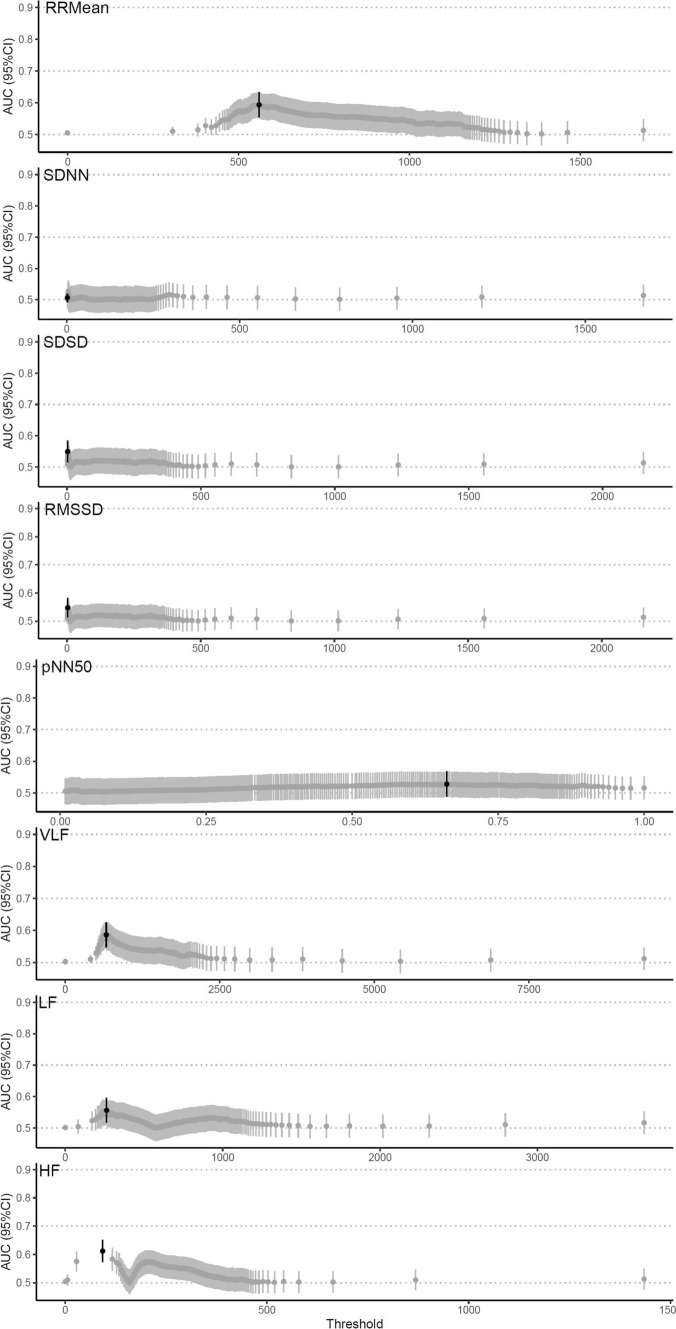
Best performing thresholds in the tertiary analysis. *AUC* area under the curve, *95% CI* 95% confidence interval, *SDNN* standard deviation of R-R intervals, *RMSSD* root mean square differences of successive R-R intervals, *RRMean* mean of R-R intervals, *SDSD* standard deviation of successive R-R interval differences, *pNN50* percentage of adjacent R-R intervals that differ from each other by more than 50 ms, *HF* high-frequency; 0.15–0.4 Hz, *LF* low-frequency; 0.04–0.15 Hz, *vLF* very-low-frequency; ≤ 0.04 Hz

When comparing the first 24 h with the last 24 h of monitoring in patients without SAEs, the optimal thresholds demonstrated low prognostic ability including analysis of the subgroups. HF demonstrated the largest point estimate of AUC 0.61 (0.57–0.65) (Fig. 4). Detailed results from the tertiary analysis are presented in Supplemental Table 4.

## Discussion

### Summary of findings

In this study, predicting SAEs based on the accumulated time below threshold values for individual HRV parameters demonstrated overall low prognostic ability. RRMean was the overall best performing parameter, having the highest AUC point-estimate across the most thresholds for both any and specific SAEs. Certain HRV measures had moderate prognostic ability for specific SAEs. In the medical subgroup, thresholds for all-cause mortality, cardiovascular, infectious, and neurologic SAEs had moderate prognostic ability when comparing the last 24 h before an SAE with the last 24 h of measurements in those without SAE. When comparing the last 24 h with the 24 to 48 h of measurements before an SAE, RMSSD, RRMean, and SDSD had moderate prognostic ability for predicting any SAEs in all patients and the surgical subgroup. The best performing thresholds in both subgroups had moderate or high prognostic ability for all specific SAEs except other and infectious SAEs in the surgical subgroup, but the limited number of SAEs challenged the statistical power. When comparing HRV measurements during the first 24 h to the last 24 h of monitoring in patients without development of SAEs, all thresholds and parameters demonstrated low prognostic ability or limited discriminative ability. This indicated minimal changes in HRV during the monitoring period for patients without development of an SAE.

### Comparisons with previous studies

In the medical subgroup, parameters for all-cause mortality (RMSSD, SDSD, pNN50, HF, SDNN, RRMean, LF) and cardiovascular SAEs (RMSSD, SDSD, pNN50, LF, SDNN) had moderate prognostic ability when comparing the last 24 h before an SAE with the last 24 h of measurements in those without SAE. Specifically, RMSSD, SDSD, pNN50, and LF had a lower confidence limit > 0.7 for prediction of cardiovascular SAEs. Other studies have associated reduced HRV with long-term mortality and cardiovascular SAEs in post myocardial infarction patients [14, 18, 25–27]. Specifically, in a meta-analysis by Fang et al. lower HRV was associated with a pooled hazard ratio of 2.27 (95% CI 1.72, 3.00) for all-cause mortality and 1.41 (95% CI 1.16, 1.72) for cardiovascular events [18]. As multiple parameters, reflecting all different parts of the ANS, had moderate prognostic ability [14], our study suggests that autonomic dysfunction might have predictive value for mortality and cardiovascular SAEs beyond post-myocardial infarction populations, even with our study having less statistical power due to shorter follow-up time and less included patients with these outcomes.

For infectious SAEs most HRV parameters, reflecting all different parts of the ANS [14], had moderate prognostic ability. Garrad et al. found significantly reduced LF and sympathetically mediated HRV during sepsis [19]. Likewise, Korach et al. reported a likelihood ratio of 6.47 for an LF/HF ratio < 1, indicating sympathetic failure, in patients with sepsis compared to those without sepsis [20]. The varied results may arise from Garrad et al. and Korach et al. assessing sepsis in a small number of ICU patients. In our study, sepsis and urinary tract infections were categorized as infectious SAEs, the latter being less severe and more common, could predominantly impact parasympathetic nervous system (PNS) or overall ANS activity.

HF, VLF, RMSSD, SDSD, and RRMean had moderate prognostic ability for neurologic SAEs in the medical subgroup including patients with stroke and syncope. Specifically, HF, RMSSD, and SDSD had a lower confidence limit > 0.7. Dütsch et al. found parasympathetic deficit in post-ischemic stroke patients, specifically with reduced HF [39]. Holmegard et al. found significantly lower overall HRV in patients with cardioinhibitory type of syncope compared to patients with vasopressor syncope during head-up tilt test and active standing, and cardioinhibitory patients showed dominance of sympathetic modulation [40]. This aligns with our results as HF, RMSSD, and SDSD reflecting short term suppression of PNS and overall ANS activity, had significant predictive ability [14]. Directly comparing physiological responses before and after a complication is likely unreasonable due to anticipated variations in HRV measurements.

When comparing the last 24 h with the 24 to 48 h of measurements before an SAE, RMSSD, RRMean, and SDSD had moderate prognostic ability for predicting any SAE in all patients and the surgical subgroup. Previous research proposed HRV as an indicator of surgical stress [13, 41–43]. Frandsen et al. continuously monitored HRV in total hip arthroplasty patients, revealing decreased HRV for at least the subsequent nine days [41], but comparability is limited due to the different study design and population. As multiple thresholds had moderate or high prognostic ability for nearly all specific SAE groups in both the surgical and medical subgroup, our study may indicate that HRV changes in patients experiencing an SAE could be a sensitive prediction method. The limited statistical power must be acknowledged as no thresholds had a lower confidence limit > 0.7 and the best performing thresholds had low prognostic ability for infectious and other SAEs in the surgical subgroup, which specifically had the highest number of SAEs.

The primary exposure variable of SDNN did not demonstrate superiority as the best HRV parameter for predicting SAEs. Furthermore, in contrast to previous studies, not a single threshold and cut-off for a HRV parameter stood out as the best predictor of SAE [11, 14, 25]. RRMean demonstrated the largest point estimates of AUC for multiple specific SAEs in both subgroup analyses. This HRV parameter is less investigated compared to RMSSD and SDNN [14], but the inverse relationship with heart rate allow comparison of our findings to other studies that have likewise described association between heart rate and complications [44–46], although generally in small or different populations from ours. Finally, when comparing the last 24 h with the 24 to 48 h of measurements before an SAE, the best performing thresholds for all patients and the surgical subgroup were consistently the largest thresholds investigated. This may suggest that the true optimal thresholds for SAE prediction were outside the range investigated. Similarly, previous studies have described that longer total or average exposure time with lower HRV in 24-h measurements indicate higher risk of mortality or complications [14, 26, 47]. The clinical relevance of the largest thresholds and beyond should be considered, if only a limited number of patients were exposed to them.

### Strengths and limitations

This study included a large sample size with a substantial number of both medical and surgical patients, even after exclusions due to SAEs occurring within the first 18 h of monitoring or patients not meeting requirements of ECG monitoring time. We were unable to find other studies that systematically explored the optimal thresholds for multiple time and frequency domain measures of HRV variables, and comprehensively evaluated HRV’s predictive performance for SAEs in hospitalised patients.

It is important to acknowledge the risk of type 1 errors, considering the explorative nature of this study that involves conducting multiple analyses. The population was included in prospective studies with specific inclusion and exclusion criteria, which limits the generalizability. The rather high exclusion of patients, especially within the medical population, emphasize that we cannot exclude stronger associations of HRV variables to SAE. The prognostic value of duration below thresholds for individual HRV parameters was assessed, but combining these parameters in a machine learning model could potentially enhance predictive performance.

### Perspectives

While it is recognized that traditional vital signs may not consistently demonstrate accurate predictive capability for upcoming SAEs [9, 10], the utilization of accumulated time below thresholds for individual HRV parameters, did not appear to have significant relevance for SAE prediction within our study population either. The moderate predictive capabilities for neurologic SAEs and cardiovascular SAEs suggest that future studies may describe this association more comprehensively, as have previously been done with long-term cardiovascular SAEs and mortality [18, 25]. Additional studies are required to validate the predictive capability of HRV for cardiovascular and neurologic SAEs in populations without pre-existing cardiovascular conditions or post-stroke [18, 25, 39]. The consistent predictive performance observed for multiple thresholds and HRV parameters in the surgical subgroup requires validation in larger studies with a more homogenous structure [48].

Larger studies including more patients with an SAE could demonstrate enhanced predictive performance by evaluating HRV changes preceding SAEs, as indicated by our secondary analysis comparing the last 24 h with the 24 to 48 h of measurements before an SAE. Our study indicated a potential association, but the existing uncertainty prevented us from reaching a definitive conclusion.

The traditional 24-h HRV measurement, while well-validated [14], have challenges in real-time SAE prediction, as the relevant 24-h window prior to an SAE is unknown. This method may help clinicians stratify patient risk but is less effective for immediate interventions. Integration with real-time HRV monitoring might be feasible. This approach would adaptively set thresholds based on analysis of continuous measurements, offering a more dynamic tool that could potentially allow for interventions before development of certain SAEs. This method requires much larger databases with more patients who develop SAEs during the monitoring period, due to the broad variation in pathophysiology associated with SAEs, complicating the development of a universal model capable of accurately predicting all SAEs or even SAEs within specific groups [49].

Future studies utilizing machine learning models to analyse multiple HRV parameters simultaneously or combine HRV data with other demographic variables may significantly enhance the predictive performance [49–51].

## Conclusion

Predicting SAEs based on the accumulated time below thresholds for individual continuously measured HRV parameters demonstrated overall low prognostic ability in high-risk hospitalized patients and no HRV parameter consistently demonstrated superiority. Multiple thresholds in the medical subgroup moderately predicted all-cause mortality, cardiovascular, infectious, and neurologic SAEs when comparing the last 24 h before an SAE with the last 24 h of measurements in those without SAE. Various thresholds had moderate prognostic ability, when comparing the last 24 h with the 24 to 48 h of measurements before an SAE, suggesting HRV changes over time could be a potentially sensitive method for predicting SAEs.

## Supplementary Information

Below is the link to the electronic supplementary material.

Supplementary file1 (DOCX 44 KB)

## Data Availability

No datasets were generated or analysed during the current study.

## References

[1] McQuillan P, Pilkington S, Allan A, Taylor B, Short A, Morgan G, et al. Confidential inquiry into quality of care before admission to intensive care. BMJ. 1998;316(7148):1853–8. 10.1136/bmj.316.7148.1853. 9632403 10.1136/bmj.316.7148.1853 PMC28582

[2] McGloin H, Adam SK, Singer M. Unexpected deaths and referrals to intensive care of patients on general wards. Are some cases potentially avoidable? J R Coll Phys Lond. 1999;33(3):255–9. PMC9665643 10402575

[3] Smith MEB, Chiovaro JC, O’Neil M, Kansagara D, Quiñones AR, Freeman M, et al. Early warning system scores for clinical deterioration in hospitalized patients: a systematic review. Ann Am Thorac Soc. 2014;11(9):1454–65. 10.1513/AnnalsATS.201403-102OC. 25296111 10.1513/AnnalsATS.201403-102OC

[4] Gerry S, Bonnici T, Birks J, Kirtley S, Virdee PS, Watkinson PJ, et al. Early warning scores for detecting deterioration in adult hospital patients: systematic review and critical appraisal of methodology. BMJ. 2020;369:m1501. 10.1136/bmj.m1501. 32434791 10.1136/bmj.m1501 PMC7238890

[5] Tarassenko L, Hann A, Young D. Integrated monitoring and analysis for early warning of patient deterioration. Br J Anaesth. 2006;97(1):64–8. 10.1093/bja/ael113. 16707529 10.1093/bja/ael113

[6] Duus CL, Aasvang EK, Olsen RM, Sørensen HBD, Jørgensen LN, Achiam MP, et al. Continuous vital sign monitoring after major abdominal surgery-quantification of micro events. Acta Anaesthesiol Scand. 2018;62(9):1200–8. 10.1111/aas.13173. 29963706 10.1111/aas.13173

[7] Pedersen NE, Rasmussen LS, Petersen JA, Gerds TA, Østergaard D, Lippert A. A critical assessment of early warning score records in 168,000 patients. J Clin Monit Comput. 2018;32(1):109–16. 10.1007/s10877-017-0003-5. 28238106 10.1007/s10877-017-0003-5

[8] Sun L, Joshi M, Khan SN, Ashrafian H, Darzi A. Clinical impact of multi-parameter continuous non-invasive monitoring in hospital wards: a systematic review and meta-analysis. J R Soc Med. 2020;113(6):217–24. 10.1177/0141076820925436. 32521195 10.1177/0141076820925436 PMC7439595

[9] Elvekjaer M, Rasmussen SM, Grønbæk KK, Porsbjerg CM, Jensen JU, Haahr-Raunkjær C, et al. Clinical impact of vital sign abnormalities in patients admitted with acute exacerbation of chronic obstructive pulmonary disease: an observational study using continuous wireless monitoring. Intern Emerg Med. 2022;17(6):1689–98. 10.1007/s11739-022-02988-w. 35593967 10.1007/s11739-022-02988-w

[10] Haahr-Raunkjaer C, Mølgaard J, Elvekjaer M, Rasmussen SM, Achiam MP, Jorgensen LN, et al. Continuous monitoring of vital sign abnormalities; association to clinical complications in 500 postoperative patients. Acta Anaesthesiol Scand. 2022;66(5):552–62. 10.1111/aas.14048. 35170026 10.1111/aas.14048 PMC9310747

[11] Heart rate variability. Standards of measurement, physiological interpretation, and clinical use. Task Force of the European Society of Cardiology and the North American Society of Pacing and Electrophysiology. Eur Heart J. 1996;17(3):354–81. 8737210

[12] Sztajzel J. Heart rate variability: a noninvasive electrocardiographic method to measure the autonomic nervous system. Swiss Med Wkly. 2004;134(35–36):514–22. 10.4414/smw.2004.10321. 15517504 10.4414/smw.2004.10321

[13] Ushiyama T, Nakatsu T, Yamane S, Tokutake H, Wakabayashi H, Ishimura K, et al. Heart rate variability for evaluating surgical stress and development of postoperative complications. Clin Exp Hypertens. 2008;30(1):45–55. 10.1080/10641960701813908. 18214733 10.1080/10641960701813908

[14] Shaffer F, Ginsberg JP. An overview of heart rate variability metrics and norms. Front Public Health. 2017;5:258. 10.3389/fpubh.2017.00258. 29034226 10.3389/fpubh.2017.00258 PMC5624990

[15] Parati G, Ulian L, Santucciu C, Tortorici E, Villani A, Di Rienzo M, et al. Clinical value of blood pressure variability. Blood Press Suppl. 1997;2:91–6. 9495635

[16] Stauss HM. Identification of blood pressure control mechanisms by power spectral analysis. Clin Exp Pharmacol Physiol. 2007;34(4):362–8. 10.1111/j.1440-1681.2007.04588.x. 17324151 10.1111/j.1440-1681.2007.04588.x

[17] Chen Y, Yu Y, Zou W, Zhang M, Wang Y, Gu Y. Association between cardiac autonomic nervous dysfunction and the severity of coronary lesions in patients with stable coronary artery disease. J Int Med Res. 2018;46(9):3729–40. 10.1177/0300060518778416. 29936852 10.1177/0300060518778416 PMC6135996

[18] Fang SC, Wu YL, Tsai PS. Heart rate variability and risk of all-cause death and cardiovascular events in patients with cardiovascular disease: a meta-analysis of cohort studies. Biol Res Nurs. 2020;22(1):45–56. 10.1177/1099800419877442. 31558032 10.1177/1099800419877442

[19] Garrard CS, Kontoyannis DA, Piepoli M. Spectral analysis of heart rate variability in the sepsis syndrome. Clin Auton Res. 1993;3(1):5–13. 10.1007/BF01819137. 8386574 10.1007/BF01819137

[20] Korach M, Sharshar T, Jarrin I, Fouillot JP, Raphaël JC, Gajdos P, et al. Cardiac variability in critically ill adults: influence of sepsis. Crit Care Med. 2001;29(7):1380–5. 10.1097/00003246-200107000-00013. 11445691 10.1097/00003246-200107000-00013

[21] Ellenby MS, McNames J, Lai S, McDonald BA, Krieger D, Sclabassi RJ, et al. Uncoupling and recoupling of autonomic regulation of the heart beat in pediatric septic shock. Shock. 2001;16(4):274–7. 10.1097/00024382-200116040-00007. 11580109 10.1097/00024382-200116040-00007

[22] Hoyer D, Friedrich H, Zwiener U, Pompe B, Baranowski R, Werdan K, et al. Prognostic impact of autonomic information flow in multiple organ dysfunction syndrome patients. Int J Cardiol. 2006;108(3):359–69. 10.1016/j.ijcard.2005.05.031. 15979171 10.1016/j.ijcard.2005.05.031

[23] Saab R, Wu BP, Rivas E, Chiu A, Lozovoskiy S, Ma C, et al. Failure to detect ward hypoxaemia and hypotension: contributions of insufficient assessment frequency and patient arousal during nursing assessments. Br J Anaesth. 2021;127(5):760–8. 10.1016/j.bja.2021.06.014. 34301400 10.1016/j.bja.2021.06.014

[24] Sun Z, Sessler DI, Dalton JE, Devereaux PJ, Shahinyan A, Naylor AJ, et al. Postoperative hypoxemia is common and persistent: a prospective blinded observational study. Anesth Analg. 2015;121(3):709–15. 10.1213/ANE.0000000000000836. 26287299 10.1213/ANE.0000000000000836 PMC4825673

[25] Kleiger RE, Miller JP, Bigger JT, Moss AJ. Decreased heart rate variability and its association with increased mortality after acute myocardial infarction. Am J Cardiol. 1987;59(4):256–62. 10.1016/0002-9149(87)90795-8. 3812275 10.1016/0002-9149(87)90795-8

[26] Buccelletti E, Gilardi E, Scaini E, Galiuto L, Persiani R, Biondi A, et al. Heart rate variability and myocardial infarction: systematic literature review and metanalysis. Eur Rev Med Pharmacol Sci. 2009;13(4):299–307. 19694345

[27] Bigger JT, Fleiss JL, Steinman RC, Rolnitzky LM, Kleiger RE, Rottman JN. Frequency domain measures of heart period variability and mortality after myocardial infarction. Circulation. 1992;85(1):164–71. 10.1161/01.cir.85.1.164. 1728446 10.1161/01.cir.85.1.164

[28] Elvekjaer M, Carlsson CJ, Rasmussen SM, Porsbjerg CM, Grønbæk KK, Haahr-Raunkjær C, et al. Agreement between wireless and standard measurements of vital signs in acute exacerbation of chronic obstructive pulmonary disease: a clinical validation study. Physiol Meas. 2021. 10.1088/1361-6579/ac010c. 33984846 10.1088/1361-6579/ac010c

[29] Haahr-Raunkjaer C, Skovbye M, Rasmussen SM, Elvekjaer M, Sørensen HBD, Meyhoff CS, et al. Agreement between standard and continuous wireless vital sign measurements after major abdominal surgery: a clinical comparison study. Physiol Meas. 2022. 10.1088/1361-6579/ac9fa3. 36322987 10.1088/1361-6579/ac9fa3

[30] Quan H, Li B, Couris CM, Fushimi K, Graham P, Hider P, et al. Updating and validating the Charlson comorbidity index and score for risk adjustment in hospital discharge abstracts using data from 6 countries. Am J Epidemiol. 2011;173(6):676–82. 10.1093/aje/kwq433. 21330339 10.1093/aje/kwq433

[31] Burr RL. Interpretation of normalized spectral heart rate variability indices in sleep research: a critical review. Sleep. 2007;30(7):913–9. 10.1093/sleep/30.7.913. 17682663 10.1093/sleep/30.7.913 PMC1978375

[32] ICH E6 (R2) Good clinical practice - Scientific guideline | European Medicines Agency. https://www.ema.europa.eu/en/ich-e6-r2-good-clinical-practice-scientific-guideline. Accessed 5 Feb 2024.

[33] Bellomo R, Ronco C, Kellum JA, Mehta RL, Palevsky P. Acute dialysis quality initiative workgroup. Acute renal failure—definition, outcome measures, animal models, fluid therapy and information technology needs: the Second International Consensus Conference of the Acute Dialysis Quality Initiative (ADQI) group. Crit Care. 2004;8(4):204–12. 10.1186/cc2872. 10.1186/cc2872 PMC522841 15312219

[34] Singer M, Deutschman CS, Seymour CW, Shankar-Hari M, Annane D, Bauer M, et al. The third international consensus definitions for sepsis and septic shock (Sepsis-3). JAMA. 2016;315(8):801–10. 10.1001/jama.2016.0287. 26903338 10.1001/jama.2016.0287 PMC4968574

[35] Thygesen K, Alpert JS, Jaffe AS, Chaitman BR, Bax JJ, Morrow DA, et al. Fourth universal definition of myocardial infarction (2018). J Am Coll Cardiol. 2018;72(18):2231–64. 10.1016/j.jacc.2018.08.1038. 30153967 10.1016/j.jacc.2018.08.1038

[36] Robin X, Turck N, Hainard A, Tiberti N, Lisacek F, Sanchez JC, et al. pROC: an open-source package for R and S+ to analyze and compare ROC curves. BMC Bioinform. 2011;12(1):77. 10.1186/1471-2105-12-77 PMC3068975 21414208

[37] Swets JA. Measuring the accuracy of diagnostic systems. Science. 1988;240(4857):1285–93. 10.1126/science.3287615. 3287615 10.1126/science.3287615

[38] Youden WJ. Index for rating diagnostic tests. Cancer. 1950;3(1):32–5. 15405679 10.1002/1097-0142(1950)3:1<32::aid-cncr2820030106>3.0.co;2-3

[39] Dütsch M, Burger M, Dörfler C, Schwab S, Hilz MJ. Cardiovascular autonomic function in poststroke patients. Neurology. 2007;69(24):2249–55. 10.1212/01.wnl.0000286946.06639.a7. 18071145 10.1212/01.wnl.0000286946.06639.a7

[40] Holmegard HN, Benn M, Kaijer M, Haunsø S, Mehlsen J. Differences in autonomic balance in patients with cardioinhibitory and vasodepressor type of reflex syncope during head-up tilt test and active standing. Scand J Clin Lab Invest. 2012;72(4):265–73. 10.3109/00365513.2012.659282. 22332835 10.3109/00365513.2012.659282

[41] Frandsen MN, Varnum C, Foss NB, Mehlsen J, Kehlet H. Time-course of heart rate variability after total hip arthroplasty. J Clin Monit Comput. 2023. 10.1007/s10877-023-00992-9. 37052614 10.1007/s10877-023-00992-9 PMC10995030

[42] Marsch SC, Skarvan K, Schaefer HG, Naegeli B, Paganoni R, Castelli I, et al. Prolonged decrease in heart rate variability after elective hip arthroplasty. Br J Anaesth. 1994;72(6):643–9. 10.1093/bja/72.6.643. 8024911 10.1093/bja/72.6.643

[43] Frandsen MN, Huang L, Petersen RH, Foss NB, Mehlsen J, Kehlet H. Continuous perioperative heart rate variability monitoring in video-assisted thoracoscopic surgery lobectomy-a pilot study. J Clin Monit Comput. 2023;37(4):1071–9. 10.1007/s10877-023-01016-2. 37243951 10.1007/s10877-023-01016-2 PMC10372135

[44] Oh H, Lee K, Seo W. Temporal patterns of change in vital signs and cardiac arrest risk triage scores over the 48 hours preceding fatal in-hospital cardiac arrest. J Adv Nurs. 2016;72(5):1122–33. 10.1111/jan.12897. 26768904 10.1111/jan.12897

[45] Candel BG, Duijzer R, Gaakeer MI, Ter Avest E, Sir Ö, Lameijer H, et al. The association between vital signs and clinical outcomes in emergency department patients of different age categories. Emerg Med J. 2022;39(12):903–11. 10.1136/emermed-2020-210628. 35017189 10.1136/emermed-2020-210628 PMC9691829

[46] Hawthorne G, Richardson M, Greening NJ, Esliger D, Briggs-Price S, Chaplin EJ, et al. A proof of concept for continuous, non-invasive, free-living vital signs monitoring to predict readmission following an acute exacerbation of COPD: a prospective cohort study. Respir Res. 2022;23(1):102. 10.1186/s12931-022-02018-5. 35473718 10.1186/s12931-022-02018-5 PMC9044843

[47] Zuanetti G, Neilson JM, Latini R, Santoro E, Maggioni AP, Ewing DJ. Prognostic significance of heart rate variability in post-myocardial infarction patients in the fibrinolytic era. The GISSI-2 results. Gruppo Italiano per lo Studio della Sopravvivenza nell’ Infarto Miocardico. Circulation. 1996;94(3):432–6. 10.1161/01.cir.94.3.432. 8759085 10.1161/01.cir.94.3.432

[48] Frandsen MN, Mehlsen J, Foss NB, Kehlet H. Preoperative heart rate variability as a predictor of perioperative outcomes: a systematic review without meta-analysis. J Clin Monit Comput. 2022;36(4):947–60. 10.1007/s10877-022-00819-z. 35092527 10.1007/s10877-022-00819-z PMC9293802

[49] Aasvang EK, Meyhoff CS. The future of postoperative vital sign monitoring in general wards: improving patient safety through continuous artificial intelligence-enabled alert formation and reduction. Curr Opin Anaesthesiol. 2023;36(6):683–90. 10.1097/ACO.0000000000001319. 37865847 10.1097/ACO.0000000000001319

[50] Deo RC. Machine learning in medicine. Circulation. 2015;132(20):1920–30. 10.1161/CIRCULATIONAHA.115.001593. 26572668 10.1161/CIRCULATIONAHA.115.001593 PMC5831252

[51] Rasmussen SS, Grønbæk KK, Mølgaard J, Haahr-Raunkjær C, Meyhoff CS, Aasvang EK, et al. Quantifying physiological stability in the general ward using continuous vital signs monitoring: the circadian kernel density estimator. J Clin Monit Comput. 2023;37(6):1607–17. 10.1007/s10877-023-01032-2. 37266711 10.1007/s10877-023-01032-2 PMC10651555

