# Supplementary material for: Prognostic value of heart rate variability for risk of serious adverse events in continuously monitored hospital patients
Source: J Clin Monit Comput. 2024 Aug 20;38(6):1315–29. doi: 10.1007/s10877-024-01193-8 (PMC11604769; doi:10.1007/s10877-024-01193-8)
Supplement: Supplementary file 1 — Supplementary file1 (DOCX 44 KB) [file 10877_2024_1193_MOESM1_ESM.docx]

Journal of Clinical Monitoring and Computing - supplementary material

Prognostic value of heart rate variability for risk of serious adverse events in continuously monitored hospital patients

Nikolaj Aagaard – Markus Harboe Olsen - Oliver Wiik Rasmussen – Katja K. Grønbæk – Jesper Mølgaard - Camilla Haahr-Raunkjaer – Mikkel Elvekjaer –Eske K. Aasvang – Christian S. Meyhoff

Corresponding author

Nikolaj Aagaard
Department of Anaesthesia and Intensive Care
Copenhagen University Hospital – Bispebjerg and Frederiksberg
DK-2400 Copenhagen, Denmark
[nikolaj.aagaard.01@regionh.dk](mailto:nikolaj.aagaard.01@regionh.dk)
+45 22908811

Table of contents

[Supplemental Table 1: Inclusion and exclusion criteria for all studies 2](#_Toc165460245)

[Supplemental Table 2: HRV-derived measures with definitions and physiological interpretations 3](#_Toc165460246)

[Supplemental Table 3: Best performing thresholds for the secondary analysis in the medical and surgical subgroup 4](#_Toc165460247)

[Supplemental Table 4: Best performing thresholds for the tertiary analysis 5](#_Toc165460248)

[References 6](#_Toc165460249)

# Supplemental Table 1: Inclusion and exclusion criteria for all studies

| Study | Inclusion criteria | Exclusion criteria |
| --- | --- | --- |
| Medical RCT  NCT04661748  H-20033246 | - Randomization possible within 12 hours - Adult patients (≥18 years) - At least one expected overnight stay - High-risk medical admission* | - Inability to cooperate or give informed consent - Allergy to plaster or silicone - Admitted for palliative care - Admission to telemetry unit - Previously enrolled in a WARD-study - A pacemaker or ICD device |
| Surgical RCT  NCT04640415  H-20034555 | - Adult patients (≥50 years - Major abdominal, orthopaedic, urologic, or vascular surgery - Duration of surgery ≥2 hours - At least two expected overnight stays - Randomization possible within 24 hours from ward arrival | - Inability to cooperate or give informed consent - Allergy to plaster or silicone - Impaired cognitive function or MMSE score < 24 - Admitted for palliative care - Admission to telemetry unit - Previously enrolled in a WARD-study - A pacemaker or ICD device |
| Medical observational study  NCT03660501  H-18026653 | - Adult patients (≥18 years) - Admission for AECOPD - Initiation of monitoring within 24 hours | - Inability to cooperate - Allergy to plaster or silicone - A pacemaker or ICD device |
| Surgical observational study  NCT03491137  H-17033535 | - Adult patients (≥60 years) - Elective major abdominal cancer surgery - estimated incision time of ≥2 hours | - Inability to cooperate or give informed consent - Allergy to plaster or silicone - Impaired cognitive function or MMSE score < 24 - Admitted for palliative care - Admission to telemetry unit - A pacemaker or ICD device |
| WARD CGM NCT04473001 H-20002220 | - Adult patients (≥18 years) - Major abdominal, orthopaedic, or vascular surgery - estimated incision time of ≥1 hour - At least one expected overnight stay - No DM, type 1, or type 2 DM | - Inability to cooperate or give informed consent - Allergy to plaster or silicone - Impaired cognitive function or MMSE score < 24 - Any treatment limitation - Previous or scheduled pancreatectomy - A pacemaker or ICD device |
| WARD VASC  NCT04628858  H-19086583 | - Adult patients (≥18 years) - PAD patients undergoing open infraligamental revascularization or bypass - At least two expected overnight stays | - Inability to cooperate or give informed consent - Allergy to plaster or silicone - Impaired cognitive function - >20 mmHg systolic BP difference between arms - Previously enrolled in a WARD-study - Any treatment limitation - A pacemaker or ICD device |
| *High-risk medical admission includes discharge from ICU-stay ≥ 24 hours or the following tentative diagnosis with at least two predefined vital sign deviations: Pneumonia, dyspnea, acute coronary syndrome, new onset heart failure, or sepsis. Legends: *RCT* Randomized controlled trial, *CGM* continuous glucose monitoring, *VASC* vascular surgery, *WARD* Wireless Assessment of Respiratory and circulatory Distress, *ICD* Implantable Cardioverter defibrillator, *MMSE* Mini-mental state examination, *AECOPD* acute exacerbation of chronic obstructive pulmonary disease, *DM* Diabetes mellitus, *PAD* peripheral arterial disease, *BP* blood pressure | | |

# Supplemental Table 2: HRV-derived measures with definitions and physiological interpretations

| Parameter | Unit | Definition | Physiological interpretation |
| --- | --- | --- | --- |
| Time-domain | | |  |
| SDNN | Milliseconds | The standard deviation of normal-to-normal R-R intervals | Reflect overall ANS activity and measure total variability [1,2] |
| RMSSD | Milliseconds | Root means square differences of consecutive R-R intervals | Reflect PNS regulation of HR and measure short-term beat-to-beat variability [1,3] |
| RRMean | Milliseconds | Mean of the R-R intervals | Inversely related to heart rate |
| SDSD | Milliseconds | The standard deviation of the consecutive  R-R interval differences | Related to SDNN but only measures short-term variability [4] |
| pNN50 | Percentage | Percentage of the consecutive  R-R intervals that differ more than 50 ms in relation to all the R-R intervals | Reflect PNS activity and measure short-term variability [1,2] |
| Frequency-domain | | |  |
| vLF | Normalized units | Normalized power of the very-low-frequency band (≤0.04Hz) | Mechanisms responsible for activity are uncertain [5] |
| LF | Normalized units | Normalized power of the low-frequency band (0.04-0.15Hz) | Reflects PNS, SNS, and baroreceptor activity [4] |
| HF | Normalized units | Normalized power of the high-frequency band (0.15–0.4 Hz) | Reflect PNS activity [6] |
| Legends:  *R-R intervals* time difference between two R peaks of two consecutive heartbeats in the ECG, *Hz* Hertz, *ms* milliseconds, *HR* heart rate, *ANS* Autonomic nervous system *PNS* parasympathetic nervous system, *SNS* sympathetic nervous system | | | |

# Supplemental Table 3: Best performing thresholds for the secondary analysis in the medical and surgical subgroup

| Type of SAE | HRV parameter | Number of SAEs | Threshold | AUC (95%CI) | Cut-off | Sensitivity (95%CI) | Specificity (95%CI) |
| --- | --- | --- | --- | --- | --- | --- | --- |
| **Medical subgroup*** | | | | | | | |
| Any | VLF | 18 | 506.69 | 0.62 (0.5-0.74) | 3.0 | 0 (0-0.19) | 0.78 (0.52-0.94) |
| Any | RRMean | 18 | 1430.98 | 0.65 (0.46-0.85) | 1439.5 | 0.61 (0.36-0.83) | 0.89 (0.65-0.99) |
| Any | RMSSD | 18 | 1465.02 | 0.66 (0.47-0.86) | 1439.5 | 0.61 (0.36-0.83) | 0.89 (0.65-0.99) |
| Any | SDNN | 18 | 1437.98 | 0.66 (0.47-0.86) | 1439.5 | 0.61 (0.36-0.83) | 0.89 (0.65-0.99) |
| Any | SDSD | 18 | 1463.85 | 0.66 (0.47-0.86) | 1439.5 | 0.61 (0.36-0.83) | 0.89 (0.65-0.99) |
| Any | HF | 18 | 706.95 | 0.67 (0.47-0.87) | 1439.5 | 0.61 (0.36-0.83) | 0.94 (0.73-1) |
| Any | LF | 18 | 1914.31 | 0.67 (0.48-0.87) | 1439.5 | 0.61 (0.36-0.83) | 0.94 (0.73-1) |
| Any | pNN50 | 18 | 0.94 | 0.7 (0.5-0.9) | 1439.5 | 0.61 (0.36-0.83) | 1 (0.81-1) |
| Respiratory SAE | VLF | 9 | 503.73 | 0.72 (0.55-0.89) | 2.0 | 0 (0-0.34) | 0.56 (0.21-0.86) |
| Other SAE | RRMean | 3 | 490.16 | 0.83 (0.51-1) | 0.5 | 0.67 (0.09-0.99) | 1 (0.29-1) |
| **Surgical subgroup** | | | | | | | |
| Any | pNN50 | 171 | 1.00 | 0.67 (0.61-0.73) | 1439.5 | 0.64 (0.57-0.71) | 0.88 (0.83-0.93) |
| Any | LF | 171 | 3561.26 | 0.68 (0.62-0.74) | 1439.5 | 0.66 (0.58-0.73) | 0.88 (0.83-0.93) |
| Any | HF | 171 | 1356.35 | 0.69 (0.63-0.75) | 1439.5 | 0.68 (0.61-0.75) | 0.88 (0.83-0.93) |
| Any | SDNN | 171 | 1589.49 | 0.69 (0.63-0.75) | 1439.5 | 0.68 (0.61-0.75) | 0.88 (0.82-0.92) |
| Any | VLF | 171 | 9016.81 | 0.69 (0.63-0.75) | 1439.5 | 0.69 (0.61-0.76) | 0.88 (0.82-0.92) |
| Any | RMSSD | 171 | 2074.59 | 0.7 (0.64-0.76) | 1439.5 | 0.69 (0.61-0.76) | 0.88 (0.82-0.92) |
| Any | SDSD | 171 | 2069.19 | 0.7 (0.64-0.76) | 1439.5 | 0.69 (0.61-0.76) | 0.88 (0.82-0.92) |
| Any | RRMean | 171 | 1633.24 | 0.71 (0.65-0.77) | 1439.5 | 0.71 (0.63-0.77) | 0.88 (0.83-0.93) |
| All-cause mortality | LF | 12 | 1463.81 | 0.81 (0.61-1) | 1439.5 | 0.83 (0.52-0.98) | 0.92 (0.62-1) |
| All-cause mortality | pNN50 | 12 | 1.00 | 0.81 (0.61-1) | 1439.5 | 0.75 (0.43-0.95) | 0.92 (0.62-1) |
| All-cause mortality | RMSSD | 12 | 456.44 | 0.81 (0.61-1) | 1439.5 | 0.83 (0.52-0.98) | 0.92 (0.62-1) |
| All-cause mortality | SDSD | 12 | 456.32 | 0.81 (0.61-1) | 1439.5 | 0.83 (0.52-0.98) | 0.92 (0.62-1) |
| All-cause mortality | VLF | 12 | 1989.46 | 0.81 (0.61-1) | 1439.5 | 0.83 (0.52-0.98) | 0.92 (0.62-1) |
| Cardiovascular SAE | SDNN | 13 | 14.96 | 0.75 (0.56-0.95) | 142.5 | 0.15 (0.02-0.45) | 0.38 (0.14-0.68) |
| Infectious SAE | RRMean | 48 | 1559.62 | 0.69 (0.57-0.8) | 1439.5 | 0.65 (0.49-0.78) | 0.92 (0.8-0.98) |
| Neurologic SAE | VLF | 4 | 1041.60 | 0.81 (0.43-1) | 52.5 | 1 (0.4-1) | 0.75 (0.19-0.99) |
| Respiratory SAE | RRMean | 19 | 1201.56 | 0.81 (0.65-0.97) | 1439.5 | 0.79 (0.54-0.94) | 0.95 (0.74-1) |
| Other SAE | RRMean | 75 | 1563.87 | 0.69 (0.6-0.78) | 1439.5 | 0.71 (0.59-0.81) | 0.85 (0.75-0.92) |
| *Legends: SAE: serious adverse events, HRV: heart rate variability, AUC: area under the curve, 95% CI: 95% confidence interval, SDNN: standard deviation of R-R intervals, RMSSD: root mean square differences of successive R-R intervals, RRMean: mean of R-R intervals, SDSD standard deviation of successive R-R interval differences, pNN50: percentage of adjacent R-R intervals that differ from each other by more than 50 ms, HF: high-frequency; 0.15-0.4 Hz, LF: low-frequency; 0.04-0.15 Hz, vLF: very-low-frequency; ≤0.04 Hz.*  **Only specific SAE outcomes analysing more than two patients were included in the table* | | | | | | | |

**Description:** The secondary analysis included only patients with an SAE after at least 48 hours of measurements and compared the last 24 hours of measurements before an SAE to the period measured 24 to 48 hours prior to the SAE. The table specifically presents the results from the subgroup analyses of medical and surgical patients.

# Supplemental Table 4: Best performing thresholds for the tertiary analysis

| Group | HRV parameter | Number | Threshold | AUC (95%CI) | Cut-off | Sensitivity (95%CI) | Specificity (95%CI) |
| --- | --- | --- | --- | --- | --- | --- | --- |
| All | SDNN | 383 | 1.69 | 0.51 (0.49-0.52) | 2.5 | 0.02 (0.01-0.04) | 0.97 (0.95-0.98) |
| All | pNN50 | 383 | 0.66 | 0.53 (0.49-0.57) | 1433.5 | 0.44 (0.39-0.49) | 0.49 (0.44-0.54) |
| All | RMSSD | 383 | 2.71 | 0.55 (0.51-0.58) | 0.5 | 0.39 (0.34-0.44) | 0.71 (0.66-0.76) |
| All | SDSD | 383 | 2.70 | 0.55 (0.52-0.58) | 0.5 | 0.39 (0.34-0.44) | 0.71 (0.66-0.76) |
| All | LF | 383 | 262.38 | 0.56 (0.52-0.6) | 1.5 | 0.56 (0.51-0.61) | 0.57 (0.52-0.62) |
| All | RRMean | 383 | 559.88 | 0.59 (0.55-0.63) | 1.5 | 0.69 (0.64-0.74) | 0.48 (0.42-0.53) |
| All | VLF | 383 | 663.01 | 0.59 (0.55-0.63) | 0.5 | 0.66 (0.61-0.7) | 0.5 (0.45-0.56) |
| All | HF | 383 | 92.74 | 0.61 (0.57-0.65) | 0.5 | 0.49 (0.44-0.54) | 0.21 (0.17-0.26) |
| Medical subgroup | RMSSD | 63 | 1.65 | 0.5 (0.46-0.54) | 1.5 | 0.06 (0.02-0.15) | 0.95 (0.87-0.99) |
| Medical subgroup | SDSD | 63 | 1.65 | 0.5 (0.46-0.54) | 1.5 | 0.06 (0.02-0.15) | 0.95 (0.87-0.99) |
| Medical subgroup | pNN50 | 63 | 1.00 | 0.54 (0.45-0.63) | 1428.5 | 0.68 (0.55-0.79) | 0.19 (0.1-0.31) |
| Medical subgroup | SDNN | 63 | 1.95 | 0.54 (0.51-0.57) | 0.5 | 0 (0-0.06) | 0.92 (0.82-0.97) |
| Medical subgroup | LF | 63 | 327.34 | 0.61 (0.51-0.71) | 66.5 | 0.4 (0.28-0.53) | 0.33 (0.22-0.46) |
| Medical subgroup | RRMean | 63 | 619.04 | 0.62 (0.53-0.72) | 243.5 | 0.41 (0.29-0.54) | 0.33 (0.22-0.46) |
| Medical subgroup | VLF | 63 | 980.75 | 0.63 (0.53-0.72) | 1265.5 | 0.13 (0.06-0.23) | 0.65 (0.52-0.77) |
| Medical subgroup | HF | 63 | 274.23 | 0.64 (0.55-0.74) | 950.5 | 0.52 (0.39-0.65) | 0.19 (0.1-0.31) |
| Surgical subgroup | pNN50 | 320 | 0.66 | 0.53 (0.49-0.57) | 1433.5 | 0.45 (0.39-0.5) | 0.48 (0.43-0.54) |
| Surgical subgroup | SDNN | 320 | 4.20 | 0.54 (0.5-0.58) | 0.5 | 0.38 (0.33-0.44) | 0.71 (0.66-0.76) |
| Surgical subgroup | RMSSD | 320 | 2.73 | 0.56 (0.52-0.6) | 0.5 | 0.42 (0.36-0.47) | 0.71 (0.66-0.76) |
| Surgical subgroup | SDSD | 320 | 2.72 | 0.56 (0.52-0.6) | 0.5 | 0.42 (0.36-0.47) | 0.71 (0.66-0.76) |
| Surgical subgroup | LF | 320 | 261.51 | 0.58 (0.54-0.62) | 2.5 | 0.5 (0.44-0.55) | 0.66 (0.61-0.71) |
| Surgical subgroup | HF | 320 | 84.04 | 0.62 (0.58-0.66) | 0.5 | 0.44 (0.39-0.5) | 0.23 (0.19-0.28) |
| Surgical subgroup | VLF | 320 | 698.74 | 0.62 (0.58-0.66) | 5.5 | 0.59 (0.53-0.64) | 0.61 (0.56-0.67) |
| Surgical subgroup | RRMean | 320 | 559.42 | 0.63 (0.59-0.67) | 20.5 | 0.45 (0.39-0.51) | 0.78 (0.73-0.82) |
| *Legends: SAE: serious adverse events, HRV: heart rate variability, AUC: area under the curve, 95% CI: 95% confidence interval, SDNN: standard deviation of R-R intervals, RMSSD: root mean square differences of successive R-R intervals, RRMean: mean of R-R intervals, SDSD standard deviation of successive R-R interval differences, pNN50: percentage of adjacent R-R intervals that differ from each other by more than 50 ms, HF: high-frequency; 0.15-0.4 Hz, LF: low-frequency; 0.04-0.15 Hz, vLF: very-low-frequency; ≤0.04 Hz.* | | | | | | | |

**Description:** The tertiary analysis compared the first 24 hours to the last 24 hours of HRV measurements in patients without an SAE, to investigate differences between the start and end of the monitoring period.

# References

1. Sztajzel J. Heart rate variability: a noninvasive electrocardiographic method to measure the autonomic nervous system. Swiss Med Wkly. 2004 Sep 4;134(35–36):514–22. DOI: https://doi.org/10.4414/smw.2004.10321

2. Umetani K, Singer DH, McCraty R, Atkinson M. Twenty-four hour time domain heart rate variability and heart rate: relations to age and gender over nine decades. J Am Coll Cardiol. 1998 Mar 1;31(3):593–601. DOI: https://doi.org/10.1016/s0735-1097(97)00554-8

3. Shaffer F, McCraty R, Zerr CL. A healthy heart is not a metronome: an integrative review of the heart’s anatomy and heart rate variability. Front Psychol. 2014;5:1040. DOI: https://doi.org/10.3389/fpsyg.2014.01040

4. Shaffer F, Ginsberg JP. An Overview of Heart Rate Variability Metrics and Norms. Front Public Health. 2017;5:258. DOI: https://doi.org/10.3389/fpubh.2017.00258

5. Kleiger RE, Stein PK, Bigger JT. Heart rate variability: measurement and clinical utility. Ann Noninvasive Electrocardiol. 2005 Jan;10(1):88–101. DOI: https://doi.org/10.1111/j.1542-474X.2005.10101.x

6. Taralov ZZ, Terziyski KV, Kostianev SS. Heart Rate Variability as a Method for Assessment of the Autonomic Nervous System and the Adaptations to Different Physiological and Pathological Conditions. Folia Med (Plovdiv). 2015;57(3–4):173–80. DOI: https://doi.org/10.1515/folmed-2015-0036
